# Potent Sensitisation of Cancer Cells to Anticancer Drugs by a Quadruple Mutant of the Human Deoxycytidine Kinase

**DOI:** 10.1371/journal.pone.0140741

**Published:** 2015-10-20

**Authors:** Safiatou T. Coulibaly, Paola Rossolillo, Flore Winter, Franziska K. Kretzschmar, Mélanie Brayé, Darren P. Martin, Daniela Lener, Matteo Negroni

**Affiliations:** 1 Architecture et Réactivité de l'ARN, CNRS, IBMC, Université de Strasbourg, 15 rue René Descartes, 67084 Strasbourg, Cedex, France; 2 Centre for High-Performance Computing, Rosebank, Cape Town, South Africa; 3 Institute of Infectious Disease and Molecular Medicine, University of Cape Town, Cape Town, South Africa; Institut National de la Santé et de la Recherche Médicale, FRANCE

## Abstract

Identifying enzymes that, once introduced in cancer cells, lead to an increased efficiency of treatment constitutes an important goal for biomedical applications. Using an original procedure whereby mutant genes are generated based on the use of conditional lentivector genome mobilisation, we recently described, for the first time, the identification of a human deoxycytidine kinase (dCK) mutant (G12) that sensitises a panel of cancer cell lines to treatment with the dCK analogue gemcitabine. Here, starting from the G12 variant itself, we generated a new library and identified a mutant (M36) that triggers even greater sensitisation to gemcitabine than G12. With respect to G12, M36 presents an additional mutation located in the region that constitutes the interface of the dCK dimer. The simple presence of this mutation halves both the IC50 and the proportion of residual cells resistant to the treatment. Furthermore, the use of vectors with self-inactivating LTRs leads to an increased sensitivity to treatment, a result compatible with a relief of the transcriptional interference exerted by the U3 promoter on the internal promoter that drives the expression of M36. Importantly, a remarkable effect is also observed in treatments with the anticancer compound cytarabine (AraC), for which a 10,000 fold decrease in IC50 occurred. By triggering the sensitisation of various cancer cell types with poor prognosis to two commonly used anticancer compounds M36 is a promising candidate for suicide gene approaches.

## Introduction

Inducing death of cancer cells through gene therapy is a major goal for biomedical applications. The main obstacle to this achievement is the possibility of delivering specifically into cancer cells transgenes that lead to cell death: a process achieved with poor efficiency *in vivo* [1]. An additional difficulty is constituted by the efficiency at which the transgene induces the death of the modified cell. Cell death can be induced either constitutively or in response to exposure to chemical compounds, such as an anticancer drug [1]. The approach of using a gene with an inducible toxicity has the advantage of being of interest also in the field of safety genes that allow negative selection of transplanted cells in gene therapy approaches. In these cases, the presence of a safety gene is essential in the event of a neoplastic transformation after engraftment of the engineered cells or, for adoptive immunotherapy, in the case of the development of a graft versus host response [2]. The suicide gene most frequently employed so far for these purposes has been the thymidine kinase gene of herpes simplex virus (hsTK) coupled with gancyclovir treatment [3–5]. However, due to its viral origin, the hsTK presents the drawback of inducing an immune response to TK-derived epitopes [6], suggesting that its replacement by less immunogenic proteins could potentially increase the efficiency of this approach. For these reasons, a human enzyme would constitute an ideal candidate.

In this regard, human deoxycytidine kinase (dCK) has attracted a lot of interest. Besides being involved in the salvage pathway that converts recycled deoxyribonucleosides into dNTP [7], this enzyme catalyzes the first rate-limiting, phosphorylation step for the activation of different deoxycytidine analogs (dCa) used in clinical treatments of various cancers. Gemcitabine is one of these drugs. It is generally used for the treatment of several cancers such as pancreatic cancer, metastatic non-small cell lung and breast cancers, as well as ovarian cancers; all of which are associated with poor prognosis [8–13]. The dCK is also involved in the activation of other compounds, that are structurally related to deoxycytidine and are used as anti-cancer or anti-viral drugs. One such drugs is AraC (cytosine arabinoside), which is mostly used for the treatment of leukaemias, such as acute myeloid leukaemia (AML) [14].

In many cases the dCK determines the degree of sensitivity of cells to treatment with gemcitabine or AraC. A correlation between the level of dCK expression and sensitivity to treatment with dCa has in fact been observed in patients treated for hairy-cell leukaemia and chronic lymphocytic leukaemia [15] and in various breast cancer cell lines [16]. A link between the expression of dCK, the accumulation and retention of intracellular gemcitabine pool levels and gemcitabine incorporation into DNA has also been reported [17–19], as well as an association between pre-treatment levels of intracellular dCK and sensitivity to gemcitabine [20]. A similar correlation has been reported for AraC [21]. Moreover, the exogenous over expression of dCK in glioma cells by retroviral or adenoviral vectors leads to increased sensitivity to the cytotoxic action of cytosine arabinoside *in vitro* and *in vivo* [22]. Conversely, deficiencies in dCK activity lead to resistance to treatment either with gemcitabine [18, 23, 24] or with AraC [25–27].

Thereby, the possibility of foreseeing gene therapy approaches based on the insertion of extra copies of the dCK gene in cancer cells appears promising and the possibility of generating variants of dCK with improved dCa phosphorylation activity is a major goal in the field. Rational attempts to design dCK mutants with such properties have been made in the past, either based on structural information [28] or on the effect of post-translational modifications of the protein that have been observed to enhance its enzymatic activity *in vivo* [29, 30]. Although these studies succeeded in obtaining dCK variants with an improved capacity to phosphorylate and activate gemcitabine, this activity was paralleled by a much more marked enhancement of phosphorylation of the natural substrate dC [28, 30]. Since competition for the dCK enzyme between natural substrate and incoming drug molecules is crucial for the efficiency of cell death induction [31, 32], this feature suggested that such mutants once introduced into cancer cells would not have induced a sensitisation to the treatment. For one of these (the triple mutant A100V, R104M, D133A) [28], it was indeed reported that its introduction into Messa10K cells, uterine sarcoma cells resistant to gemcitabine [33], resulted in no sensitisation to this drug [34].

We recently developed an experimental approach (retrovolution) where a gene of interest is inserted into conditional replication-defective VSV-pseudotyped HIV-1 derived vectors that are used to mimic multiple infectious cycles, during which the sequence of interest accumulates mutations due to the error-prone nature of the HIV-1 replication machinery [34]. This generates a library of variants of the gene of interest that are already inserted into a biological vector appropriate for an efficient delivery of the transgene to human cells, which can then be directly screened for the desired phenotype. Using, as a sequence of interest, the cDNA coding for the human dCK, we isolated a mutant, named G12, which induces a 300-fold sensitisation to gemcitabine in cells originally resistant to this drug (Messa 10K), an effect 60 times stronger than that observed in controls cells where an extra copy of the wt dCK had been inserted [34]. This mutant is characterised by the presence of three mutations, located in positions of the protein distant from the active site and which are therefore unlikely to have been predicted on a rational basis. One of these mutations, E171K, is located in a region involved in the generation of the interface of the dCK dimer. The other two mutations (E247K and L249M) instead are located in the “base-sensing loop” that modulates the folding of the protein upon binding of the phosphate donor (ATP or UTP), (S1 Fig) [28]. No alteration of the degree of dimerization was however observed *in vitro* for the mutant, suggesting that the reason for the increased gemcitabine sensitivity that it induces is not attributable to a change in its expression expression level [34], but is possibly due to a modification of the overall structure of the enzyme.

G12 was isolated after screening of the library resulting from 17 successive cycles of retrovolution [34]. This way, mutations were introduced randomly in the gene with no external selection pressure applied during the procedure that would favour the isolation of the desired phenotype. The question of the possibility of isolating a mutant with an even greater degree of gemcitabine sensitisation activity than G12 is the starting point of the present study.

## Materials and Methods

### Cell lines

HEK-293T cells were obtained from the American Type Culture Collection and grown in Dulbecco’s Modified Eagle’s Medium (Gibco, Thermo Fischer Scientific, Waltham, MA, USA) supplemented with 10% FBS and 100U/ml pennicillin-100 mg/ml streptomycin. Messa10K cells were kindly provided by L.P.Jordheim (Lyon, France) and grown in RPMI medium supplemented with 10% FBS and penicillin-streptomycin.

### Retrovolution cycles by lentivector particles

For starting the experiments leading to the generation of the F27-RL (where RL stands for random library), eight 10-cm plates containing 5x10^6^ cells from the population of HEK 293T cells that was used to identify the G12 clone (F16, reference [34]) were transfected, using the Calcium Phosphate protocol, with 10 μg of pCMV∆R8.91 plasmid [35] and 5 μg of pHCMV-G plasmid [36], in order to generate the viral-like population F17-RL. The same procedure was used for starting the experiments leading to the generation of the F11-DL, but in this case the initial transfection with pCMV∆R8.91 and pHCMV-G plasmids was performed on a HEK 293T cell line previously generated to stably express the lentivector genomic RNA coding G12 [34]. In this case the viral-like population generated was called F1-DL (where DL stands for directed library). These viral-like populations (F17-RL and F1-DL) were then used to independently transduce fresh HEK 293T cells to continue with the retrovolution procedure in parallel, as described in reference [34]. We performed 11 further retrovolution cycles, leading to the generation of the F27-RL and of the F11-DL cell populations, respectively. Transfections during the cycles of retrovolution were achieved with 10 μg of pCMV∆R8.91 and 5 μg of pHCMV-G plasmid on 8 10-cm plates containing 5x10^6^ cells from the population of HEK 293T cells from the previous retrovolution cycle. For the transduction procedure during the cycles of retrovolution, the lentivector-containing supernatant was collected 48h after transfection, filtered through a 0.45 μm filter and concentrated 40 times in Vivaspin 20 columns (MWCO 50 KDa, Sartorius Stedim Biotech, Aubagne, France). Transduction conditions differed when performed to generate genetic diversity (retrovolution cycles) and when performed to isolate single clones for screening of the library. For the retrovolution cycles, 5x10^6^ HEK-293T cells were transduced with 1 ml of concentrated lentiviral vectors (see above) and puromycin selection was applied 24h after transduction. Under these conditions 90–100% of the transduced cells were resistant to puromycin, indicating that at least for the majority of the cells, the multiplicity of transduction was higher than one. Transductions for the isolation of single clones were, instead, performed at varying dilutions of the lentiviral vectors based on the titers estimated by ELISA directed against the HIV-1 p24 capsid protein (Innotest HIV Antigen mAb, Innogenetics, Gent, Belgium). A hundred cells were then seeded per well in 96-well plates in DMEM. Puromycin selection was applied 24h after transduction by adding puromycin at concentrations of 0.6 μg/ml for HEK-293T cells, 0.6 μg/ml for HEK-293T-CD4^+^ cells, 0.5 μg/ml for Messa10K cells. We selected conditions where, on average, 5 wells per plate resulted positive to cell growth in the presence of puromycin. Under these conditions, the multiplicity of transduction was of 5x10^-4^ per cell ensuring that in each well the cell population was generated by transduction with a single lentiviral vector.

### Amplification and sequencing of the dCK-encoding portion of the proviral DNA

Puromycin resistant clones from the F27-RL and F11-DL generations were isolated by limiting dilution and expanded in 6 wells plates. Cells from the individual clones were then recovered and lysed with 250 μl of Cell Direct PCR (Viagen Biotech, Los Angeles, CA, USA). The dCK transgene was amplified from 1 μl of the lysate with oligonucleotides on the vector, EF1 (5’-gatgtcgtgtactggctccg-3’) and PGK (5-gatgtggaatgtgtgcgagg-3’), flanking the transgene. Sequencing of the PCR fragment was performed by the GATC sequencing service (GATC Biotech, Konstanz, Germany).

### Calculation of the frequency of mutations in the libraries

For the F27-RL and F11-DL libraries the number of mutations found in the dCK coding sequence was calculated starting from the data given in Table 1 as: (m/(783 x c))/y, where m is the number of mutated positions, 783 is the size of the dCK coding-sequence in base pairs, c is the number of clones sequenced and y is the number of cycles of retrovolution carried out. For the F11-DL library the starting sequence for calculating the mutation rates was the sequence of G12.

**Table 1:** Mutation panel found in the dCK coding-sequence in F27-RL and in F10-DL.

	Clones sequenced	Positions mutated	Frequency of mutation (per nt)	% of G>A mutations	Mutations/ clone	Cycles of retrovolution
**F27-RL**	41	57	6.34x10^-5^	70.9	1–11	28
**F11-DL**	43	29	7.83x10^-5^	75.9	1–5	11

Frequencies of mutation are calculated as described in Materials and Methods; %G>A mutations: % of transitions from G to A among the number of mutated positions found; mutations/clone: range of mutations found in single clones (for the F10-DL library the three mutations present in G12 are not considered).

### Permutation tests for the occurrence of synonymous and non synonymous mutations along the dCK protein

A permutation test was performed by simulating the occurrence of mutations in the two halves of the protein coding sequence (i.e. on either side of codon 130) based on the observed frequency of occurrence of random mutations and of preferential mutations in APOBEC3F consensus sequences (29.1 and 70.9%, respectively, see Table 1). The relative incidence of synonymous and non-synonymous mutations obtained experimentally was compared to that of 10,000 simulated datasets. The proportion of simulated datasets with non synonymous/synonymous (dN/dS) mutation ratios lower than or equal to those of the real dataset were then taken as the probability that there was a significant tendency in the region considered for the occurrence of more synonymous mutations than was expected on a stochastic basis. Conversely, the proportion of simulated datasets with dN/dS ratios higher than or equal to those of the real dataset was then taken as the probability that there was a significant tendency in the region considered for the occurrence of more non-synonymous mutations than was expected on a stochastic basis.

### Tests of sensitivity to anticancer drugs

Cells harbouring integrated lentiviral vector genomes (G12, M36, single or double mutants) were seeded at 5 000 cells per well in a 96-well plate and grown overnight at 37°C. For each population experiments were carried out in duplicate. Twelve hours after seeding, increasing concentrations of gemcitabine (0–400 nM), or AraC (0–10 mM) were added to each well and left in culture for a further 72h. Cell viability was measured using the MTT test (CellTiter 96 Non-Radioactive Cell Proliferation Assay, Promega, Fitchburg, WI, USA) and the number of living cells in each well was evaluated by measuring the OD at 570 nm. For each population, the fraction of living cells was calculated as OD570 concentration X Gem/OD570 concentration 0 Gem, to estimate the sensitivity of the population to the prodrug.

### Tests of sensitivity to antiviral drugs

Lentiviral vectors were generated by transfection, using the calcium phosphate protocol, of HEK-293T cells with 10 μg of pCMV ∆R8.91 plasmid [35], 5 μg of pHCMV-G plasmid [36], and 10 μg of genomic plasmid SDY-dCK (described in Rossolillo et al. [34]) in its SIN version (see results) encoding either for the wild type sequence of the human dCK or the M36 variant. Several transductions, using different amounts of lentiviral vector, were performed on 1x10^6^ HEK-293T-CD4+ cells [37] and transduced cells were selected in the presence of 0.6 μg/ml of puromycin, added 24h after transduction. Only transduction conditions giving 50 to 200 individual puromycin-resistant clones (each clone derived from a cell transduced by a single lentiviral vector) were retained and all the clones were pooled and expanded to obtain a polyclonal HEK-293T-CD4+ cell population either expressing one extra copy of the wt dCK enzyme or one copy of M36 (HEK 293T/CD4^+^-SINvec-wtdCK or HEK 293T/CD4^+^-SINvec-M36, respectively). Vectors for monitoring the efficiency of transduction in the presence of antiviral drugs were generated by transfection of twelve 10-cm plates containing 5x10^6^ HEK 293T cells with 10 μg of pTopo plasmid into which the sequence coding for the HIV-1 envelope ADA [38] had been inserted [39], and 10 μg of pNL4.3-Env^—^Luc+, that encodes the firefly luciferase, as previously described [40]. The resulting lentivectors were concentrated as previously described [34]. Transduction of 2.5x10^5^ HEK 293T/CD4^+^-SINvec-wtdCK cells and HEK 293T/CD4^+^-SINvec-M36 cells were performed in parallel using 1 ml each of concentrated lentiviral vector. Five hours after transduction cells were seeded at 5x10^4^ cells/well in a 6 well plate in the presence of increasing concentrations of the anti-viral compounds ddC or 3TC. The efficiency of transduction was then monitored 48 hours after transduction through expression of the luciferase gene. For this, the medium was removed, the cells were washed twice in PBS, lysed, centrifuged and the supernatant was then used to measure luciferase activity according to the manufacturer’s protocol (Promega, Fitchburg, WI, USA) with a Glomax luminometer (Promega, Fitchburg, WI, USA), as previously described [40].

### Western blot analyses

Messa10K cell lines expressing either M36, both in the context of a lentiviral vector DNA with a functional LTR or with an inactivated LTR were lysed in 1X RIPA buffer (25mM Tris-HCl, 1% IGEPAL, 0,1% SDS, 0,5% Sodium deoxycholate, 150mM NaCl, protease inhibitor) and 30, 60, and 120 μg of total protein (evaluated by Bradford assay) for each cell type were loaded on a 12% bis-tricine gel (Invitrogen, Carlsbad, NM, USA). After transfer on PVDF membrane, the dCK proteins were analysed by western Blot with 1:4000 dilution of a polyclonal anti-dCK antibody (rabbit, Sigma-Aldrich, St. Louis, MO, USA) and 1:3000 dilution of an anti-rabbit HRP conjugated secondary antibody (BioRad, Hercules, CA, USA) with binding being detected by chemiluminescence (Pierce ECL Western Blotting substrate, Thermo Scientific).

### Production and purification of recombinant dCK proteins

The wt-dCK and M36 sequences were cloned in the pET14b plasmid and expressed in *E*. *coli* cells BL21 DE3 pLysE. Protein expression was induced by adding 0.1mM IPTG and cells were collected after 4h of growth at 37°C. His-tagged proteins were eluted with 250 mM imidazole from His-Trap TM FF Columns (GE Healthcare Life Science, Little Chalfont, UK), the Histidine tag was removed using the S-Tag Thrombin Purification Kit (Novagen, Madison, WI, USA), and dCK and M36 were further purified by gel filtration on S-200 Sephacryl columns (GE Healthcare Life Science, Little Chalfont, UK).

### Phosphorylation tests *in vitro*

The efficiency of phosphorylation of the natural substrate deoxycytidine and of the prodrugs gemcitabine and AraC were measured for purified wt-dCK and M36 in a NADH-based assay as previously described [41]. All reagents were purchased from Sigma-Aldrich (St. Louis, MO, USA) except gemcitabine (Lilly, Indianapolis, IN, USA). Enzymes were assayed at room temperature at a concentration of 0.9 μM for dC, and 0.3 μM for both gemcitabine and AraC. dC was used at concentrations between 5 and 50 μM, gemcitabine at concentrations between 20 μM and 1mM and AraC at concentrations between 10 μM and 0.5 mM. ATP was used at a concentration of 4 mM.

### Statistical analyses

To evaluate whether the average amounts of cell death occurring in the Messa10K populations containing different dCK variants were significantly different from the value shown by Messa10K wt-dCK, a two sample t-test was applied for the different concentrations of Gemcitabine.

## Results

### Contribution of the individual mutations of G12 to sensitisation to gemcitabine

**Fig 1:**
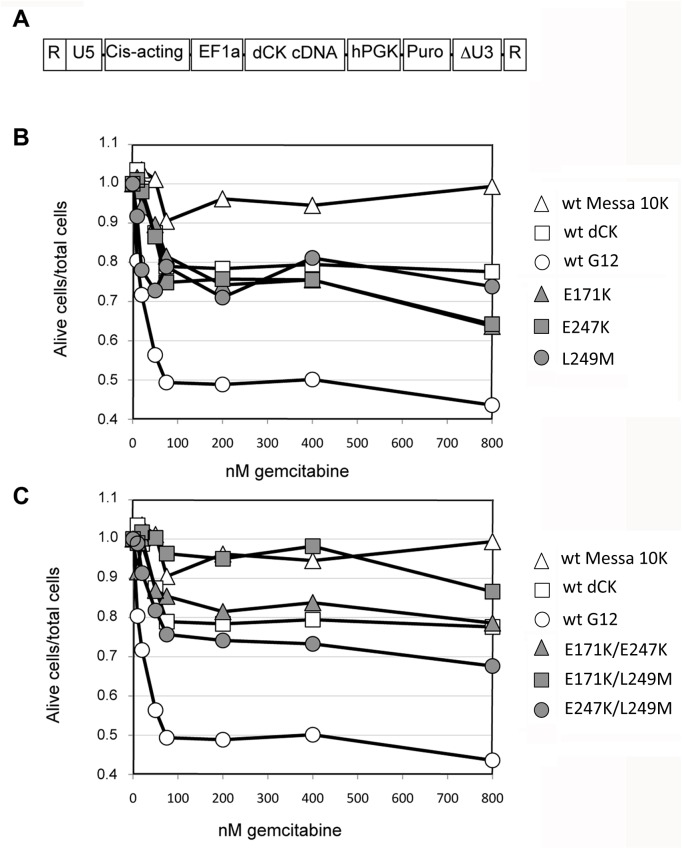
Contribution of the individual mutations of G12 to the observed phenotype. Panel A. Structure of the genomic RNA generated by transcription after transfection of cells with pSDY-SIN plasmids. R, repeated sequence from HIV-1 genome; U5, 5' unique sequence of HIV-1 LTR; Cis-acting, sequences required for packaging and reverse transcription of the genomic RNA; EF1-alpha, human elongation factor 1-alpha promoter; hPGK, human phosphoglycerate kinase promoter; Puro, puromycin N-acetyl-transferase gene; ∆U3, partially deleted version of the 3' unique sequence of HIV-1 LTR. Panels B and C. The proportion of alive cells over the total number of cells as function of the concentration in gemcitabine is given as a ratio with respect to the alive cells observed in the absence of drug. White symbols represent reference populations in both panels and. grey symbols represent single and double mutants (as described in the main text). Since single and double mutants were tested in parallel in the same experiment. The data are the average of 3 independent experiments. Error bars are not shown for the sake of clarity.

Using a new method of directed human gene evolution that we have developed in our laboratory, we previously described the isolation and characterisation of a dCK mutant, named G12, that induces sensitisation of cancer cells to treatment with the anticancer compound gemcitabine [34]. G12 is characterized by three mutations with respect to the wild type dCK enzyme. However, it has remained undetermined whether the sensitisation activity observed in G12 depends on the presence of all three mutations and, if so, what the relative contribution of each of these mutations to the G12 phenotype is. To address these issues, we constructed six mutants, three containing either the E171K, the E247K, or the L249M mutation alone, and three containing two mutations each, covering all the possible combinations of double mutants (E171K/E247K, E171K/L249M, and E247K/L249M). The mutants were inserted in the previously described pSDY plasmid [34], and used for transfection with transcomplementation plasmids as described in the Materials and Methods, in order to generate lentiviral vectors. With respect to the pSDY plasmids we previously described [34], the plasmids we used (pSDY-SIN) contained a partially deleted and therefore non-functional version of the 3' U3 sequence (Fig 1A). The resulting lentiviral vectors will generate, after reverse transcription, non-functional LTR sequences and are therefore called self-inactivating (SIN) vectors (SDY-SIN in our case). These lentivectors were chosen for this test for their pertinence for biomedical applications. As controls, the G12 and the wt dCK sequences were also inserted in pSDY-SIN plasmid. For each variant of dCK, the lentivectors were used to establish a polyclonal population of Messa 10K cells. These polyclonal populations (SINvec-G12 population for G12, SINvec-wt dCK for wt dCK, SINvec-E171K for the E171K mutants, and so on) were generated by transducing 10^6^ HEK 293T cells with different amounts of lentiviral vector in order to obtain, after puromycin selection, 50 to 200 individual clones. This way, each clone was almost certainly derived from a cell containing a single integrated copy of proviral DNA. The clones were then pooled ensuring that the resulting population was constituted by cells carrying the same transgene, but with the gene integrated at different locations in the genome, thereby averaging over possible effects that might be attributable to the integration site. The fact that the cells contained a single copy of the transgene allowed the exclusion, when comparing populations one with the other, of possible dose effects due to the number of transgene integrated. Sensitisation to gemcitabine treatment was then evaluated by MTT tests. To this end, the different Messa 10K polyclonal populations were μded into 96-well plates, exposed to varying concentrations of gemcitabine, and cell survival was measured for each condition as previously described [34].

The three single mutants (grey symbols Fig 1B) yielded response curves in the same range as the reference wt dCK population (white squares). The same results were obtained for the double mutants E171K/E247K, and E247K/L249M (grey triangles and circles in Fig 1C, respectively). All three of the single mutants and two of the three double mutants that were tested were unable to induce gemcitabine sensitisation more efficiently than was achievable with an extra copy of the wt dCK gene. The third double mutant, E171K/L249M (grey squares in Fig 1C), yielded a result similar to that observed for wt Messa 10K cells (white triangles in the Fig), suggesting that an inactivation of dCK activity occurred for this mutant, leaving the cells deprived of dCK activity (as is the case with the original Messa 10K line). In conclusion, since none of the single and double mutants displayed a response curve similar to that of G12 (white circles in the Fig), the simultaneous presence of the three mutations is apparently required to achieve the sensitisation phenotype observed with G12.

### Generation of libraries using "random" versus "directed" evolution

**Fig 2:**
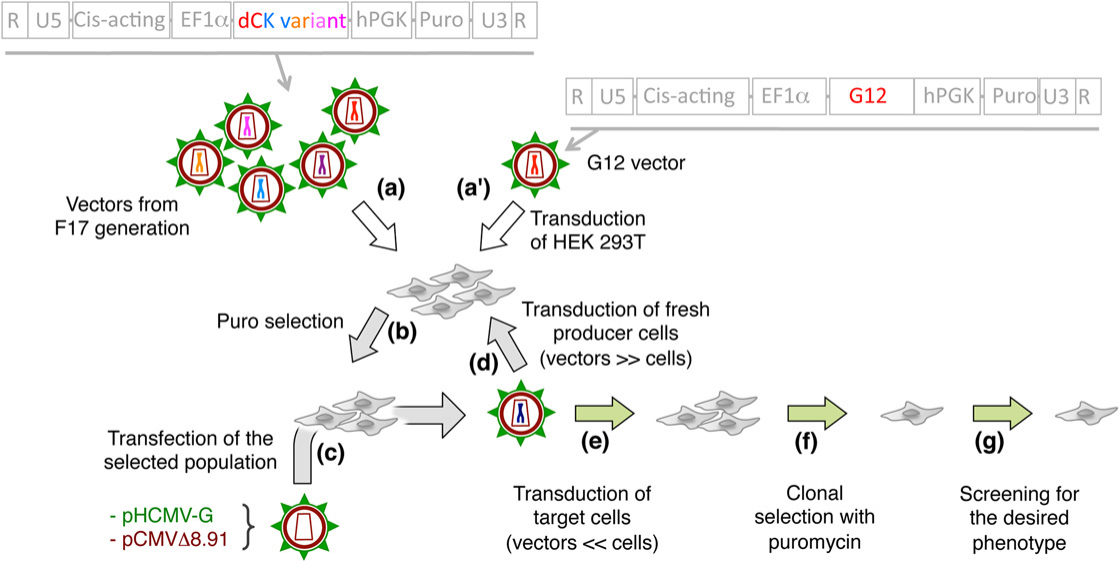
Scheme of the procedure followed to generate F27-RL and F11-DL. The general structure of the genomic RNAs used for retrovolution is given at the top, on the left for the F16 population and on the right for the population containing only the G12 variant. The procedure has been previously described [34] and consists in the repeated transduction of HEK 293T cells with the lentiviral vectors followed by the selection, with puromycin, of cell populations that have stably integrated the proviral DNA that has been produced by reverse transcription. Transfection of this population with the transcomplementation plasmids pHCMV-G and pCMV∆8.91 (see main text) allows the production of the next vector generation that is then used to transduce fresh HEK 293T cells during repeated cycles of selection, transfection and transduction. For screening purposes, target cells are transduced by less than one lentivector particle per cell, and individual clones were isolated in the presence of puromycin. Screening for sensitisation to various compounds is carried out as described in the main text.

The G12 mutant had been isolated, starting from the wt dCK sequence, from a library of dCK variants obtained after 17 generations of retrovolution (called F16, the first generation being constituted by the parental vectors P [34]). Here we investigated whether performing retrovolution starting from the G12 sequence instead of the wt dCK sequence, would yield mutants with a further improved phenotype. For this purpose, the HEK 293T cell line stably expressing G12 that we previously generated [34], was used to start 11 cycles of retrovolution, by transfection with the transcomplementation plasmids pCMV∆R 8.91 and pHCMV-G [35, 36] as described in Materials and Methods and outlined in Fig 2. This led to the generation of the population of HEK 293T cell library we call F11-DL (for F11 directed library). In parallel, in order to evaluate the possibility of isolating mutants with an improved phenotype with respect to G12 simply by continuing the retrovolution procedure that led to the isolation of G12, we also performed a further 11 cycles of retrovolution on the entire F16 population from which G12 was isolated [34], so as to reach the F27 generation (we call F27-RL, for F27 random library).

To isolate individual clones from each library, for the last cycle of retrovolution the F10-DL and F26-RL virus-like populations were used to independently transduce fresh HEK 293T cells at a multiplicity of vector particles per cell that was substantially lower than 1. Transduced cells were seeded into 96-well plates, and selection with puromycin was applied as described in Materials and Methods, in such a way that each clone contained a single copy of the transgene. Individual clones were expanded and used for sequencing analysis. For the functional screening of the libraries, the HEK 293T clones isolated by this method were transfected with the transcomplementation plasmids, and the resulting virus-like populations were used to transduce Messa 10K cells to generate polyclonal populations, as described above. The resulting populations were used for testing for the sensitivity to anticancer and antiviral compounds.

### Sequencing of the libraries

To evaluate the complexity of the library and characterise the nature of the mutations generated during the 11 cycles of retrovolution that were carried out in the DL and in the RL libraries, 41 HEK 293T clones from the F27-RL and 43 from the F11-DL were sequenced in the dCK-coding region, after PCR amplification from proviral DNA. The clones of the F27-RL library presented point mutations distributed all along the dCK gene, with 57 positions that were mutated (Table 1 and S1 Fig), resulting in a mutation frequency of 6.34 x 10^−5^ nt/cycle per site (a total of 28 cycles having been performed to create this library). G>A transitions represented 70.9% of the mutations, in line with the mutation bias of HIV-1 reverse transcriptase. Of note, the majority of these transitions fell in the typical recognition motif of APOBEC3F (GA>AA).

**Fig 3:**
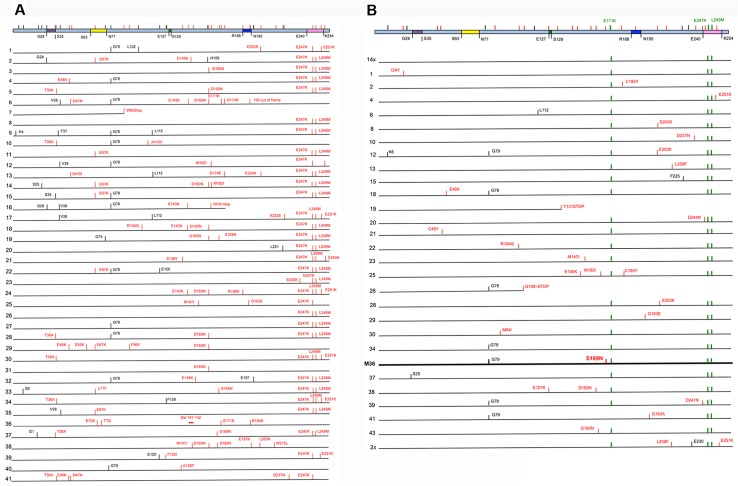
Position of the mutations found in the individual clones. In each panel, the dCK protein is represented (top of the drawing) as a pale blue box with the main structural motifs given in color (the first and last aminoacid of each motif is given below the box): purple, P-loop; yellow, insert; green, ERS; blue, lid; pink, base sensing loop; as defined in [28]. For each panel, a cumulative map of the positions that were mutated is provided at the top of the box with aminoacid substitutions given in red, while aminoacids that did not change but with associated codons that carried synonymous mutations given in black. Panels A and B respectively show the patterns observed for the random and directed libraries, respectively. Two deletion were also found: one single nucleotide deletion in clone 6 indicated as "193 out of frame", and a two-nucleotide deletion (positions 141–142) in clone 36 indicated by a red horizontal line. In Panel B, clone 36 (corresponding to M36 in the main text, for Mutant 36) is given in bold.

These mutations generated aminoacid changes all along the protein. However, while the distribution of the mutations was not different (p = 0.3657) between the two halves of the protein (aa 1–130 and 131–260) with 26 mutations in the first half, and 29 in the second, the majority of the mutations falling in the first half of the gene surprisingly corresponded to silent mutations (in black in Fig 3A). Namely, while in the first part 13 of the 25 positions mutated (52.0%) generated synonymous mutations (one mutation generating a stop codon was not considered), only 2 synonymous mutations were present among the 27 positions (7.4%) that were mutated in the second half of the protein (two mutations generating stop codons were not considered). In the first half of the protein, the ratio of non-synonymous mutations over synonymous (dN/dS) was significantly lower than expected under random mutation (p = 0.0019, as defined by permutation tests, see Material and Methods) indicating the occurrence of purifying selection on the N-ter half of the protein during the retrovolution cycles in HEK 293T cells. In sharp contrast, the C-ter half of the protein yielded dN/dS ratios indicative of the occurrence of significant (p = 0.00143) positive selection.

The analysis of the clones sequenced from the directed evolution library F11-DL (S2 Fig and Fig 3B) revealed that 14 clones out of 43 did not present additional mutations with respect to the three mutations observed in G12. The other 29 clones presented 1 to 5 additional nucleotide mutations (1 to 3 aminoacidic substitutions) for a total of 29 mutated positions, giving a mutation frequency of 7.83 x10^-5^ (Table 1). As for the RL library, most mutations (75.9%) were G>A transitions. Overall, the pattern and frequency of mutations in the two libraries were similar to one another other. Also in this case an unbalanced distribution of non-synonymous versus synonymous mutations in the two halves of the protein was observed, with 40.0% of mutations in the first half being synonymous as opposed to only 14.3% being synonymous in the second half (note that the three mutations found in G12 were not considered here since they were present in the starting sequence).

### Screening of the libraries

**Fig 4:**
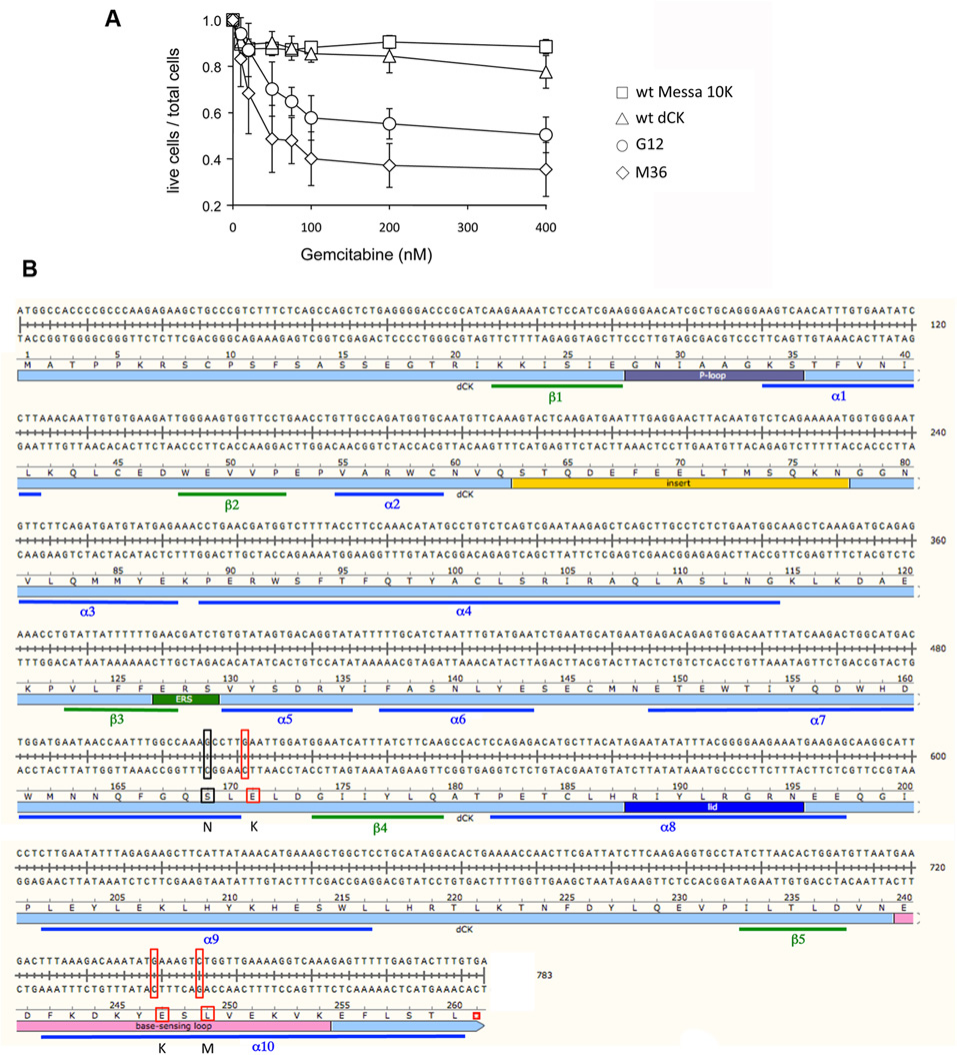
The M36 mutant. Panel A. Sensitivity to gemcitabine of Messa10K cells induced by M36. The ratio of living cells over the total number of cells is plotted as a function of the concentration of gemcitabine. Data are the average of five independent experiments. Panel B. Localisation of the mutations in the human dCK that characterise M36. The nucleotide sequence (double stranded) is given above, with the aminoacid sequence below. The full-length protein is given by a pale blue bar with the major functional domains, as defined in [28], indicated in colour.

Forty-one and twenty-three Messa 10K polyclonal populations from F27-RL and F11-DL, respectively, each derived from an individual clone of HEK 293T isolated as described above, were then screened for the induction of an increased sensitivity to the antitumor compound gemcitabine. In the F27-RL, no clones inducing sensitisation to treatment with gemcitabine were isolated. Only one clone presented the 3 G12 mutations together, but the association with other mutations generated an aberrant protein with no sensitisation phenotype. The analysis of the F11-DL library revealed that the association of the G12 mutations with the majority of the other mutations along the protein either did not change the phenotype with respect to G12 or it completely abolished sensitisation to gemcitabine. One variant, though, named M36 (represented in Fig 3B), increased Messa 10K cell sensitivity to gemcitabine to a degree greater than that of G12 (p<0.005), which was tested in parallel (Fig 4A). Besides the three mutations typical of G12, this mutant presented the additional substitution S169N (Fig 3B). Therefore, four mutations clustered in pairs in the 169–171 (S169N, E171K) and in the 247–249 (E247K, L249M) regions (Fig 4B and S3 Fig) characterise the M36 mutant isolated from F11-DL.

### Characterisation of M36 in cell culture with a ∆LTR lentiviral vector

**Fig 5:**
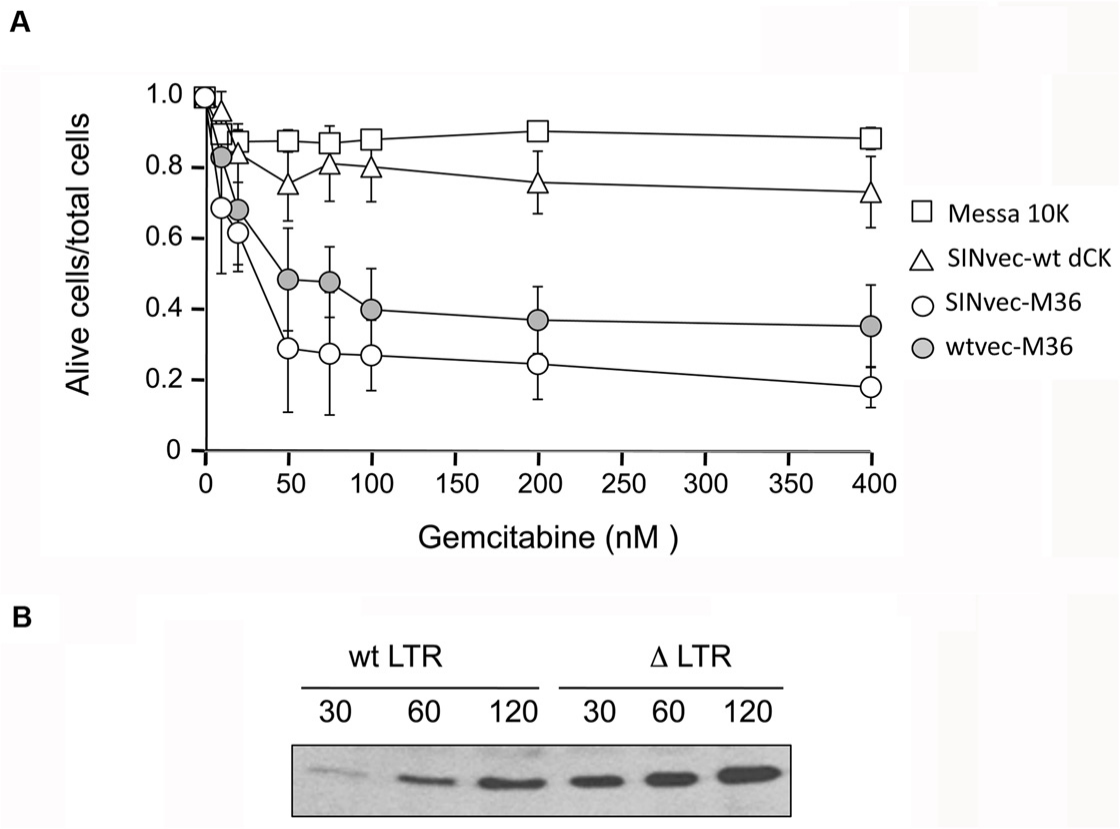
Use of a self-inactivating (SIN) LTR sequence. Panel A, sensitivity of cells harbouring a proviral DNA carrying SIN LTR and expressing M36 (white circles); of cells harbouring a proviral DNA carrying wt LTR and expressing M36 (grey circles, the data for this curve are reported as reference from Fig 4A, where they appear as white diamonds); of cells harbouring a proviral DNA carrying SIN LTR and expressing the wt dCK (white triangles); of wt Messa 10K cells (white squares). The proportion of living cells over the total number of cells is plotted as a function of the concentration of gemcitabine. The averages of three independent experiments are presented. Panel B, Western blot analysis of the expression of wt dCK and of M36 in wt-LTR and in SIN-LTR vectors. The band corresponding to the human dCK is shown for protein extracts from a population of Messa 10K cells expressing the M36 variant from a proviral DNA containing either a wt LTR or a SIN LTR. Three different amounts of total protein extract were loaded for each sample, as indicated (in μg) above each lane. The picture shows a representative result of 3 independent Western blot experiments.

The need for functional LTRs is intrinsic to the retrovolution procedure. However, as mentioned above, the use of vectors carrying a self-inactivating LTR (called SIN vectors) is required in view of prospective biomedical applications. The expression of the transgene from a proviral DNA deprived of a functional promoter for the synthesis of the viral genomic RNA could, however, be altered, leading to a change in the profile of sensitisation of the transgenic cells. To evaluate this possibility, the M36-coding sequence has been inserted in a SIN version of pSDY (Fig 1A) that has been used to generate lentiviral vectors by transfection of HEK 293T cells with the transcomplementation plasmids, as described above. These vectors have been used to establish a polyclonal population of Messa 10K cells (SINvec-M36 population), on which cell death was then measured in response to varying concentrations of gemcitabine. As a control, the survival of the SINvec-wt dCK Messa 10K polyclonal population (described above) was evaluated in parallel. As shown in Fig 5A, a strong sensitisation was observed relative to both wt Messa cells and to the SINvec-wt dCK population (white squares and white triangles in Fig 5A, respectively), in cells expressing the M36 (white circles) transgene from proviral DNA produced following reverse transcription of SIN vectors. Noteworthy, sensitisation was stronger (p<0.05) in the SINvec-M36 population than it was in a polyclonal population expressing M36 from a vector carrying a wt LTR (wtvec-M36 population reported as reference in the figure with grey circles). With respect to the wtvec-M36 population, the IC50 for the SINvec-M36 population was reduced from 50 to 24 nM. In addition, the plateau of residual survival observed was also reduced in the SINvec-M36 population, from around 40% to around 20% (Fig 5A). These efficiencies of cell death are in the range of those observed with the use of SIN vectors for combination hsTK + gangcyclovir treatment [42].

Since an increased degree of sensitisation in the SINvec-M36 population could be attributable to increased M36 production in the presence of a ∆LTR variant, we investigated by Western blot whether a higher intracellular concentration of dCK/M36 was present in the SINvec-M36 polyclonal population of Messa10K cells than in the wtvec-M36 cell population. As shown in Fig 5B, more protein was detected in SINvec-M36 cells than was detected in wtvec-M36 cells. This difference in the amount of protein could account for the increased sensitisation observed for the cells transduced with SIN vectors.

### M36 and sensitisation to antiviral compounds

**Fig 6:**
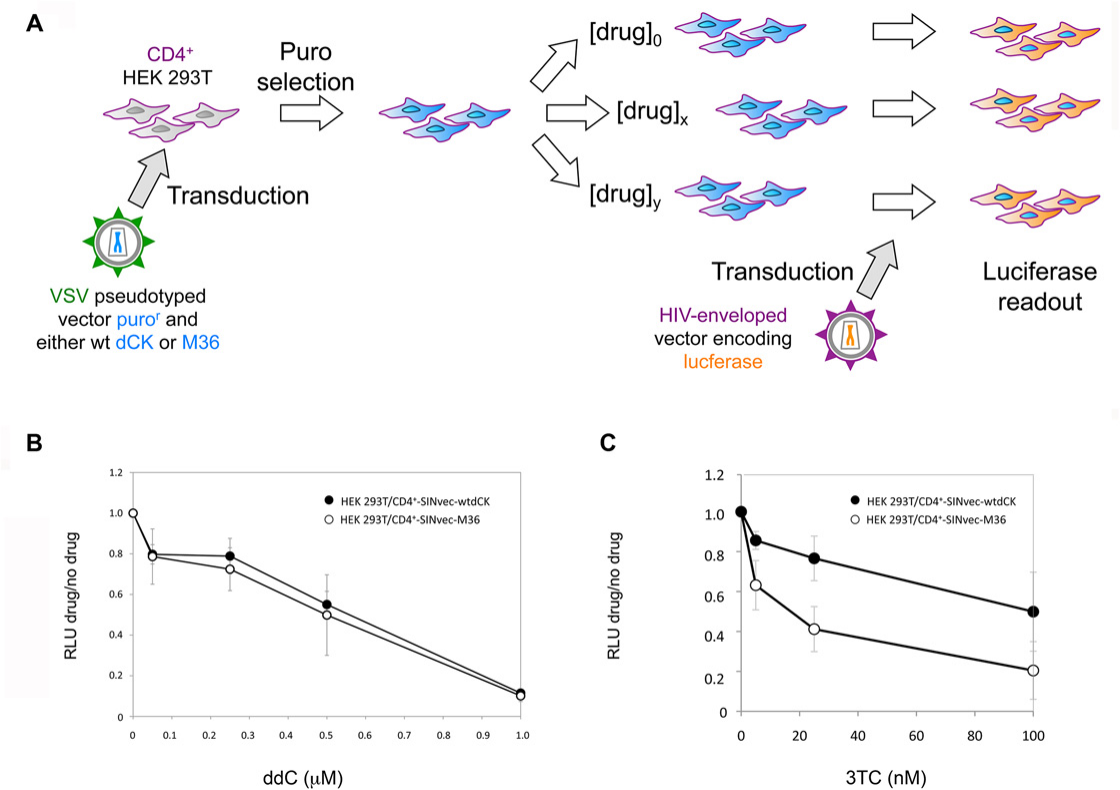
M36 and sensitisation to anti HIV drugs. Panel A. Schematic representation of the experimental procedure used to test the level of resistance to infection by HIV-derived vectors of cells expressing constitutively M36 and treated in the presence of the anti-HIV compounds ddC and 3TC. Panels B and C. Sensitivity to transduction with HIV-derived vectors in the presence of varying concentrations of ddC (panel B), or 3TC (panel C), of HEK 293T-CD4+ cells encoding M36 (white circles), or an extra copy of the wt dCK gene (black circles). Data are the average of 3 independent experiments. RLU, relative luciferase units.

We then investigated whether M36 could induce sensitisation to drugs used for antiviral treatments. Specifically, two deoxycytidine analogs used as antiviral drugs that are activated through phosphorylation by dCK were tested, 3TC and ddC. To this end, we developed the antiviral assay depicted in Fig 6A. As described in the Materials and Methods, we generated HEK 293T/CD4^+^ cell lines stably expressing either M36-SIN or wt dCK-SIN as a control; these cell lines were respectively named HEK 293T/CD4^+^-SINvec-M36 and the HEK 293T/CD4^+^-SINvec-wtdCK. For testing resistance to infection of each of these cell lines, cells were cultivated in the presence of varying concentrations of antiviral drug for 24 hours and then transduced with lentiviral vectors carrying the HIV-1 ADA envelope and encoding the luciferase gene, as detailed in the Materials and Methods and outlined in Fig 6A. Cells were maintained in the presence of the antiviral compound, and luciferase levels were measured 48 hours after transduction (Fig 6B and 6C). In cells expressing M36, no significant increase in transduction resistance was observed in the presence of ddC (Fig 6B). A significant (p<0.05) decrease in the efficiency of transduction was instead observed for 3TC (Fig 6C), for which 164 nM of 3TC was required to avoid 50% of transduction in the presence of an extra copy of dCK while only 15 nM of the drug was sufficient to reduce transduction by 50% in cells expressing M36.

### M36 and sensitisation of cells to other anticancer compounds

**Fig 7:**
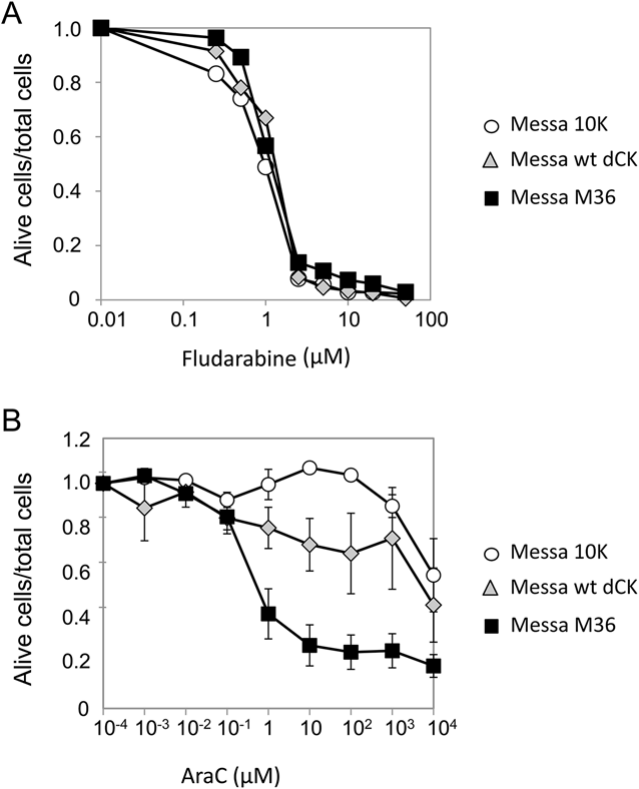
M36 and sensitisation to anticancer compounds other than gemcitabine. Sensitivity to anticancer compounds of Messa10K cells induced by M36. The percentage of living cells over the total number of cells is plotted as a function of the concentration of the anticancer compound (the x axis is given in log scale). Panel A, sensitivity to fludarabine (average of three independent experiments, error bars are not shown for the sake of clarity). Panel B, sensitivity to AraC. Data in panel B give the average of five independent experiments.

The possibility that M36 might sensitise cancer cells to anticancer compounds other than gemcitabine was then tested by evaluating sensitisation of Messa 10K cells to two compounds used in the clinical treatment of cancer that are structurally related to gemcitabine: fludarabine and AraC. The tests were performed as previously described for gemcitabine on SINvec-M36 and, as control, on SINvec-wt dCK Messa 10K polyclonal populations. Markedly different results were obtained with the two compounds. Although no sensitisation by M36 was observed (Fig 7A) in the case of fludarabine, strong sensitisation to AraC was observed in SINvec-M36 cells (Fig 7B, black squares) relative to both SINvec-wt dCK cells (grey diamonds) and to wt Messa 10K cells (white circles). A 10,000 fold decrease in IC50 (p<0.001) was observed for SINvec-M36 cells relative to SINvec-wt dCK cells.

### Biochemical characterisation of phosphorylation of gemcitabine and AraC by M36

**Fig 8:**
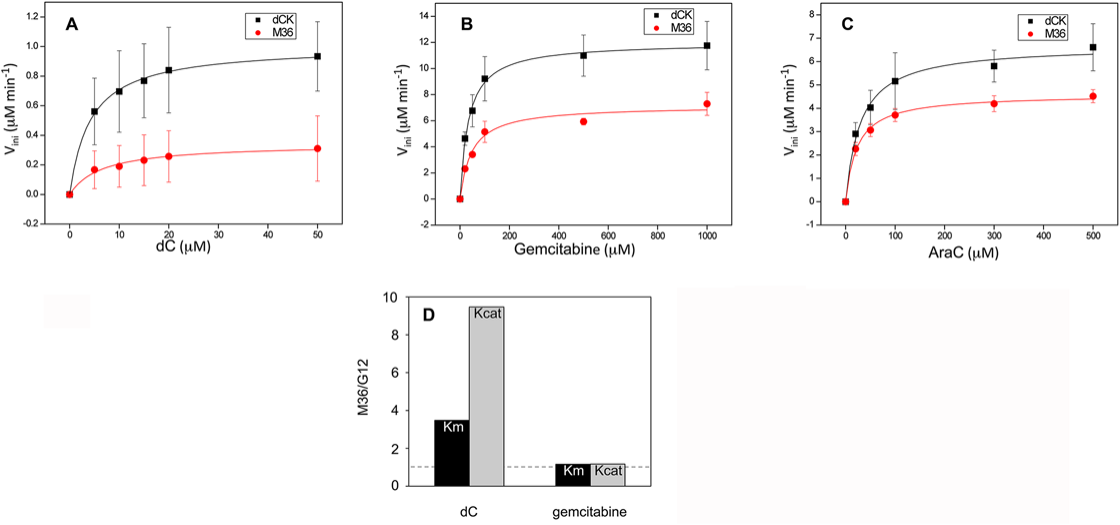
Biochemical comparative characterisation of phosphorylation of AraC by wt dCK and M36. Panels A and C. Phosphorylation kinetics of purified wt dCK (black squares) and M36 (red circles). Steady state kinetic data were fitted according to the Michaelis-Menten equation. Panel A, phosphorylation of dC (average of four independent experiments). Panel B, phosphorylation of gemcitabine (average of four independent experiments). Panel C, phosphorylation of AraC (average of three independent experiments). Panel D, ratio of Km (black) and of Kcat (grey) for M36 vs G12, with respect to dC and to gemcitabine. The dotted line gives the reference of a ratio of 1.

To characterise the biochemical properties of M36 and, as a control, of wt dCK, the two enzymes were produced as recombinant, N-terminal His tagged proteins in *E*. *coli*. The purified enzymes were tested for their efficiency at phosphorylating dC (the natural substrate), gemcitabine and AraC *in vitro*, as previously described [34]. For the three substrates, a decrease in the initial rate of phosphorylation (V_ini_) as a function of the concentration of substrate was observed for M36 with respect to the wt enzyme (red and black, respectively, in Fig 8 panels A to C) with a decrease in Vmax that was more important for dC (3-fold) than for gemcitabine and AraC (1.6 and 1.5 -fold, respectively).

A comparative analysis of the kinetic parameters of dCK and M36 with respect to phosphorylation of dC, gemcitabine and AraC is given in Table 2. While the Kcat/Km, indicative of the overall efficiency of the reaction, was 2.6 times lower for M36 than for the wt dCK when considering the natural substrate, it was only 1.3 and 1.1 lower for gemcitabine and for AraC, respectively. The decrease in efficiency of phosphorylation of M36 specifically for dC is expected to bias phosphorylation in favour of the drugs providing a likely explanation for the sensitisation of the cells that express M36 to these drugs.

**Table 2:** Kinetic parameters of the M36 mutant with respect to dC, gemcitabine and AraC.

	**dC**		
	Km *µM*	K_cat_ *s* ^*-1*^	K_cat_/Km *(M* ^*-1*^ *s* ^*-1*^ *) x 10* ^*3*^
**dCK**	5.42 ± 1.89	0.035 ± 0.010	6.46
**M36**	7.31 ± 2.11	0.018 ± 0.005	2.46
	**Gemcitabine**		
	Km *µM*	K_cat_ *s* ^*-1*^	K_cat_/Km *(M* ^*-1*^ *s* ^*-1*^ *) x 10* ^*3*^
**dCK**	46.7 ± 18.9	0.57 ± 0.15	12.21
**M36**	38.7 ± 7.69	0.37 ± 0.05	9.56
	**AraC**		
	Km *µM*	K_cat_ *s* ^*-1*^	K_cat_/Km *(M* ^*-1*^ *s* ^*-1*^ *) x 10* ^*3*^
**dCK**	28.2 ± 3.80	0.38 ± 0.08	13.48
**M36**	20.5 ± 3.76	0.25 ± 0.01	12.20

To understand the molecular bases of the M36 mediated sensitisation of cells to gemcitabine relative to that of G12, Km and Kcat values for dC and gemcitabine were compared between these two mutants by dividing the values found with M36 by those we previously reported for G12 [34]; (AraC was not tested with G12 in that work). Relative to G12, M36 has an increased Km (by a factor of 3.5, black in Fig 8D) and Kcat (by a factor of 9.5, grey in the figure) for the natural substrate dC. For gemcitabine, however, the two enzymes displayed almost identical Km and Kcat values (ratios of 1 between M36 and G12). The fact that the kinetic parameters for gemcitabine are unaltered between the two mutants suggests that the different levels of sensitisation to gemcitabine observed in cell culture must be due to differences in their relative abilities to phosphorylate the natural substrate. In this respect, the values of Kcat and those of Km of the two dCK mutants point in two opposite directions. The differences observed with dC imply that M36 has a lower affinity for the natural substrate than G12, while it has improved catalytic activity that will facilitate phosphorylation of the natural substrate compared to G12. If we compare the time needed for the transformation of one molecule of substrate into product (1/Kcat) G12 will bind 3–4 times more efficiently the natural substrate than M36, but it will require a 9–10 longer time to complete phosphorylation (G12 1/Kcat = 526 sec, M36 1/Kcat = 55 sec). These results indicate that G12 will be blocked in a non productive state that will impede the phosphorylation of the drug, while M36 will proceed to a new round of catalysis.

## Discussion

Suicide gene therapy is an approach of interest for many biomedical applications such as in the clinical treatment of cancers and as a safety gene in cell therapy. We previously described the identification of G12, a triple mutant of the human dCK that sensitises a panel of cancer cells to treatment with gemcitabine at doses significantly lower than those required for induction of wild type cell death [34]. Here, by identifying a new mutant (M36), we extend these findings in two main directions. We observed an improved sensitisation to gemcitabine with respect to G12 and we report that a dramatic sensitisation is also observed with AraC, an anticancer compound that, as with gemcitabine, is currently used in clinics for the treatment of poor prognosis cancers. Furthermore, we also report that cells expressing the transgene are resistant to transduction with HIV-1 derived vectors at concentrations of 3TC significantly lower than those required for wt cells, which is consistent with the activation of analogues of dC by M36.

The G12 mutant was identified by screening a library of dCK variants that was created by using retrovolution, a new procedure developed in our laboratory, where controlled successive cycles of transduction are carried out using conditional replication-defective HIV-1 derived viral vectors that contain the dCK coding sequence in their genomic RNA. As the number of cycles progresses, mutations accumulate in the target gene due to the error-prone nature of the HIV-1 reverse transcription process. We observed the presence of synonymous mutations at higher than expected frequencies in the N-terminus half of the protein, indicative of purifying selection acting during the retrovolution cycles. Since the cycles are performed in HEK 293T cells that contain functional dCK, it is possible that this effect is due to the removal of dominant negative mutants that might be generated in this part of the protein during the procedure. In this regard, the N-ter half of the protein encompasses a large part of the dimer interface (S4 Fig), and the mutants that are counter-selected could interfere with the correct formation of dimers of the endogenous dCK dimers that are presumably present in HEK 293T cells. In contrast, positive selection was observed in the second half of the protein, indicative of a potential advantage for the expression of dCK variants with mutations in this region. This part of the protein contains the substrate binding site as well as the phosphate donor site. It is possible that modifications in this region favoured the growth of HEK 293T cells containing the transgene, during the retrovolution cycles. However, irrespective of the underlying mechanism, documenting the occurrence of purifying selection underscores an interesting asset of this system: mutants that are toxic to the cells are removed during the procedure. Such mutants might be toxic even in the absence of the drug, and therefore engineered cells containing them would be of little use for inducing controlled cell death in response to drug treatment.

We performed 11 further cycles of retrovolution either on the entire library from which G12 was isolated (random library) or starting from the G12 sequence alone (directed library) aiming at isolating a dCK mutant with greater sensitisation activity than G12. Whereas screening of the random library failed to identify new mutants of interest, either with a genotype related to G12, or with a completely different set of mutations a mutant with a related genotype to G12 but with an improved sensitisation phenotype was isolated from the directed library. This result indicates that productively extending the mutational pathway of G12 was likely more easily achievable than was developing a mutant along a completely different mutational pathway. Although gemcitabien sensitisation conferring dCK mutants that have a totally different genotype to that of G12 and M36 are likeky possible, this might require a set of mutations that are too complex to be easily identified through retrovolution. The difficulty in obtaining a mutant conferring improved sensitisation to anticancer drug treatment is further underlined by the following considerations. One is that the analysis of the directed library revealed that in the majority of cases the association of the G12 mutations with single mutations in other regions of the protein completely abolished the gemcitabine sensitisation phenotype (data not shown). Another is the absolute need for the three mutations that characterize G12 to be present simultaneously to obtain sensitisation of cells to the drug: mutants containing only one or two of the G12 mutations failed to induce the sensitisation phenotype (Fig 1, panels B and C).

The extra mutation (S169N) characterizing M36 with respect to G12 is located two aminoacids upstream one of the three G12 mutations (E171K). This results in a protein with four mutations clustered by pairs in a region in close proximity of the surface of the protein involved in the formation of the dimer and in the "base sensing loop" region (S3 Fig). Each cluster contains one mutation that leads to a drastic change in charge (E replaced by K, for positions 171 and 247) and another pair that is conservative of the physicochemical properties of the residues involved (replacement between non-polar residues at position 249 and between polar residues at position 169). These two "mild" mutations, albeit evidently important, probably reflect fine-tuning adjustments in the local folding of the regions that harbour the more drastic changes due to the two E to K substitutions. It is noteworthy that the mild mutation that differentiates M36 and G12, S169N, removes a potential phosphorylation site, possibly impacting the activity of the enzyme altering its post-translational modifications *in vivo*. It should however be stressed that phosphorylation of S169, if it indeed occurs, is probably not relevant for the activity of the enzyme since the characterisation of the dCK phosphorylation sites has identified T3, S11, S15, and, more importantly, S74 but not S169 as phosphorylation sites *in vivo* [43]. Therefore, loss of the potential phosphorylation site at S169 is unlikely to be responsible of the differences in activity observed between M36 and G12.

A feature emerging from the biochemical characterisation of M36 is that the mutant displays, both with respect to the wild type enzyme and to G12, markedly modified kinetic parameters relative to the natural substrate dC but only mild changes with respect to either gemcitabine or AraC. Indeed, the efficiency of phosphorylation (expressed as Kcat/Km values) for gemcitabine and for AraC is not significantly different between M36 and wt dCK (Table 2) while the mutant phosphorylates the natural substrate four times less efficiently than wt dCK. As a result, M36 phosphorylates the drugs with increased efficiency relative to the natural substrate. Decreased ability to phosphorylate the natural substrate seems to be the preferred path to inducing increased sensitivity to anticancer drugs. In support of this view, an alteration in its capacity to phosphorylate the natural substrate also seems to explain the improved phenotype of M36 with respect to G12: relative to M36, G12 appears to both bind the natural substrate more avidly, and takes longer periods once bound before it releases the phosphorylated molecule, and therefore has lower availability for binding the drug molecules.

Besides the fact that increases in sensitisation to drug treatment that are induced by G12 and M36 are strongly impacted by the efficiencies with which these mutants hosphorylate the natural substrate, the characteristics of specific drug molecules are also likely highly relevant. Indeed, while M36 increases cellular sensitivity to both gemcitabine and AraC (for which the effect is markedly stronger than for gemcitabine), it failed to detectably sensitise cells to the other anticancer drug tested, fludarabine (an analogue of dA), an observation that might be accounted for by the fact that dA is not the preferential substrate of dCK [44]. In addition to activating anticancer drugs, M36 also activated one of the two antiviral compounds tested, 3TC, effectively protecting M36-containing cells from transduction with HIV-1 derived vectors at 3TC doses eleven times lower than those required to protect wt cells. The lack of effect observed with ddC is probably due to this molecule being too structurally dissimilar to its natural substrate.

We also report here that the use of the SIN vectors for inserting M36 into the target cells, instead of vectors carrying a wt LTR, further sensitises cells to gemcitabine exposure. This is probably due to the relief of transcriptional interference that is expected to occur with the functional U3 promoter [45]. In support to such a view, we detected higher intracellular quantities of synthesised M36, when the protein was expressed from a proviral DNA derived from a SIN vector than when it was synthetised from a wt vector. In line with the reported direct correlation between the amounts of dCK protein that are present in a cell and the degree to which a cell is sensitive to anticancer compounds [21], such vector-mediated increases in cellular concentrations of sensitisation mutant protein such as M36, should lead to an increased intracellular concentration of activated drug molecules and therefore increased degrees of cellular.

In conclusion, these results describe the identification and molecular characterisation of a new variant of the human dCK enzyme that could be of great interest as either a safety gene with utility in cell therapy approaches, or as suicide gene for cancer treatment. The ability of M36 to strongly sensitise cells to two of the drugs that are routinely employed in the treatment of different types of cancer with poor prognoses makes this dCK mutant a particularly promising candidate for suicide gene approaches.

## Supporting Information

S1 Fig Schematic representation of the nucleotide substitutions generated in the 41 clones of the F27-RL. Colour code: red G>A; green C>T; violet T>C; light blue C>A; pink T>A; dark blue A>C; black A>T. A cumulative map of the positions mutated along the protein is given at the top of the figure. The light blue segments indicate regions of 100 nt.
(TIF)

S2 Fig Schematic representation of the nucleotide substitutions generated in the 43 clones of the F11-DL. The representation and colour code is the same as in S1 Fig.
(TIF)

S3 Fig Schematic diagram of a dCK monomer. The G12 mutations are outlined in red and the additional M36 mutation is outlined in yellow.
(TIF)

S4 Fig Structure of a human dCK dimer. The part in blue constitutes the N-ter half of the protein; in grey the C-ter half and in red highlighted the positions mutated in M36.
(TIF)
