# Supplementary figures and images for: Potent Sensitisation of Cancer Cells to Anticancer Drugs by a Quadruple Mutant of the Human Deoxycytidine Kinase
Source: PLoS One. 2015 Oct 20;10(10):e0140741. doi: 10.1371/journal.pone.0140741 (PMC4618062; doi:10.1371/journal.pone.0140741)

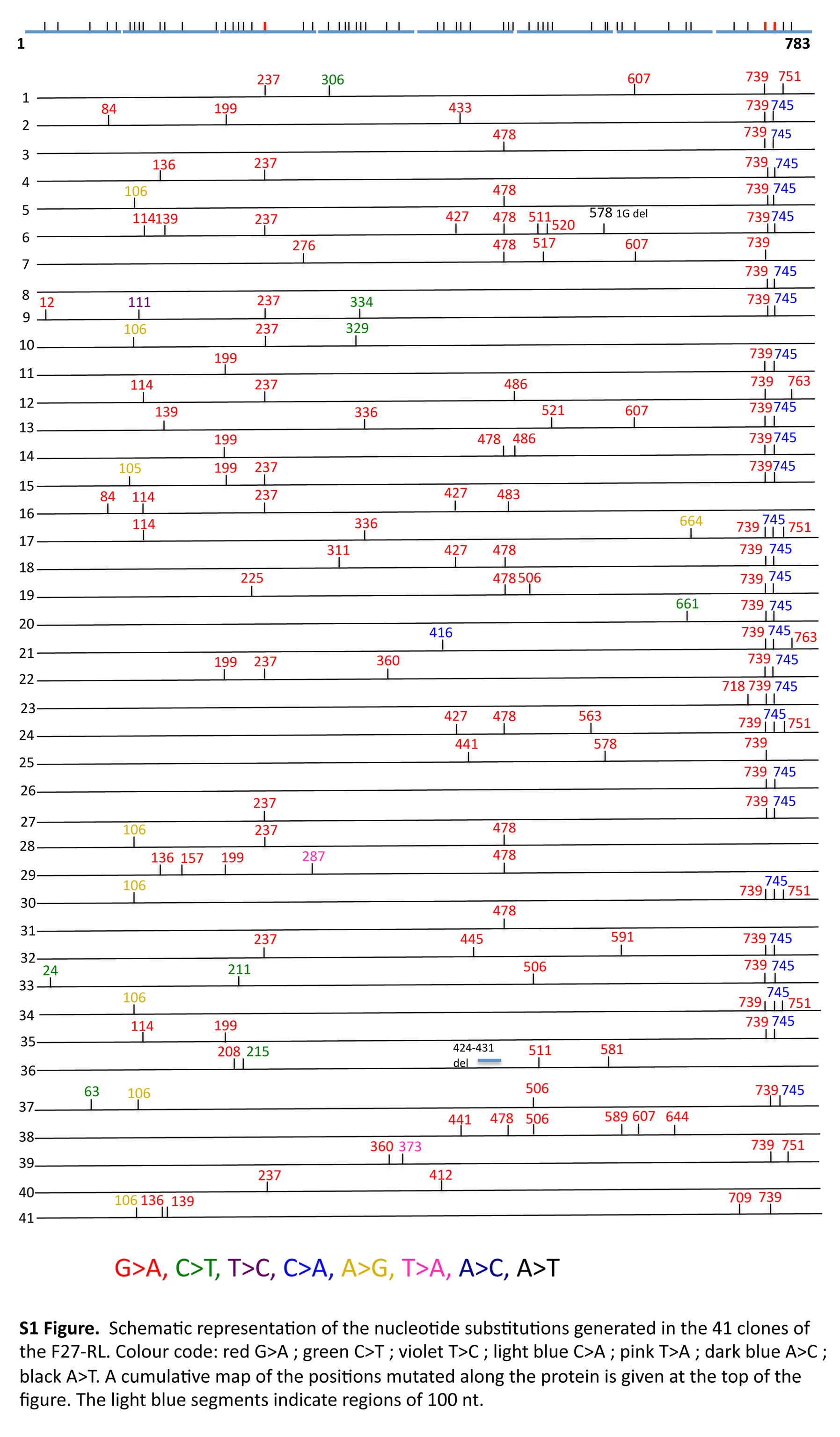

Supplement: S1 Fig — Colour code: red G>A; green C>T; violet T>C; light blue C>A; pink T>A; dark blue A>C; black A>T. A cumulative map of the positions mutated along the protein is given at the top of the figure. The light blue segments indicate regions of 100 nt. (TIF) [file pone.0140741.s001.tif]

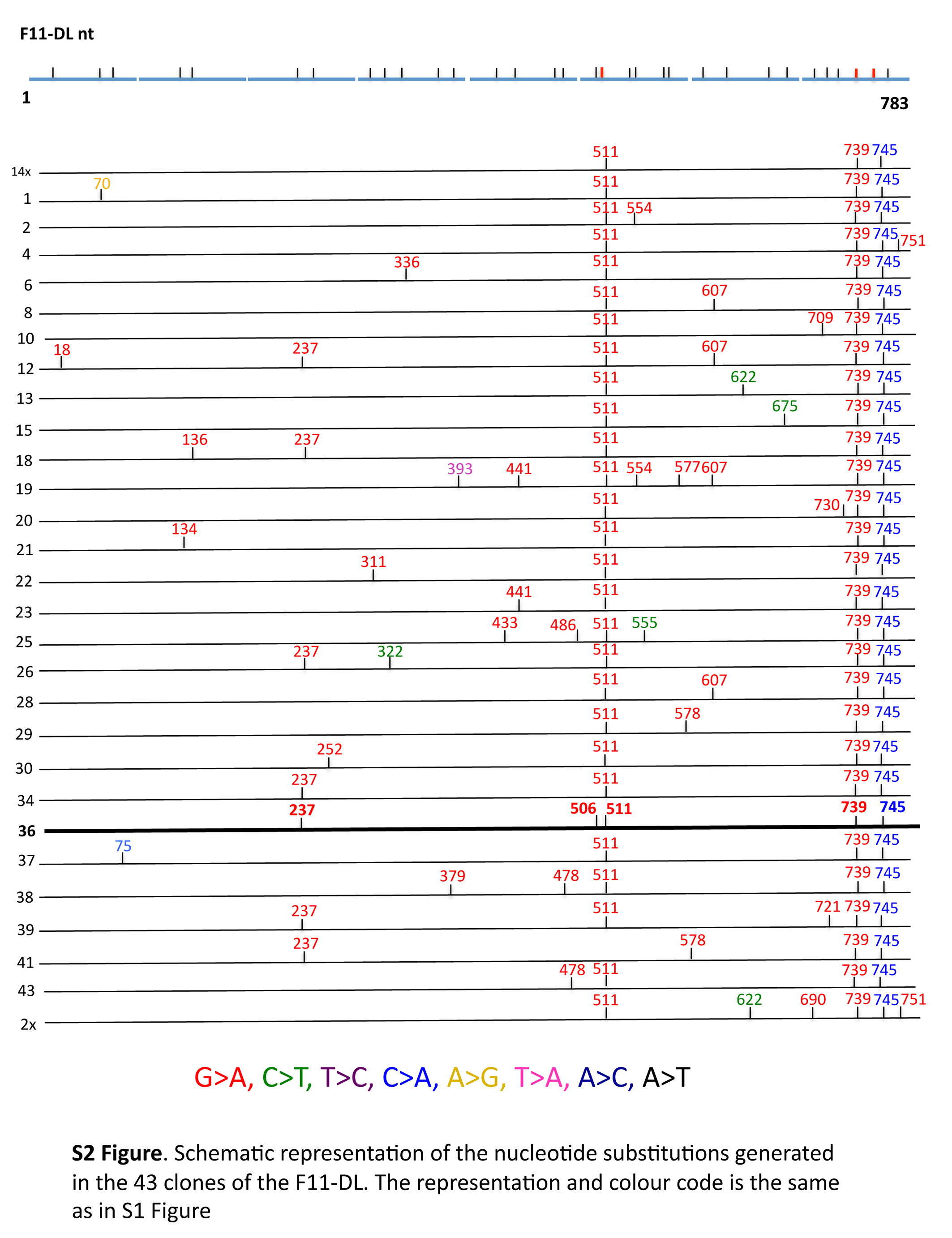

Supplement: S2 Fig — The representation and colour code is the same as in S1 Fig. (TIF) [file pone.0140741.s002.tif]

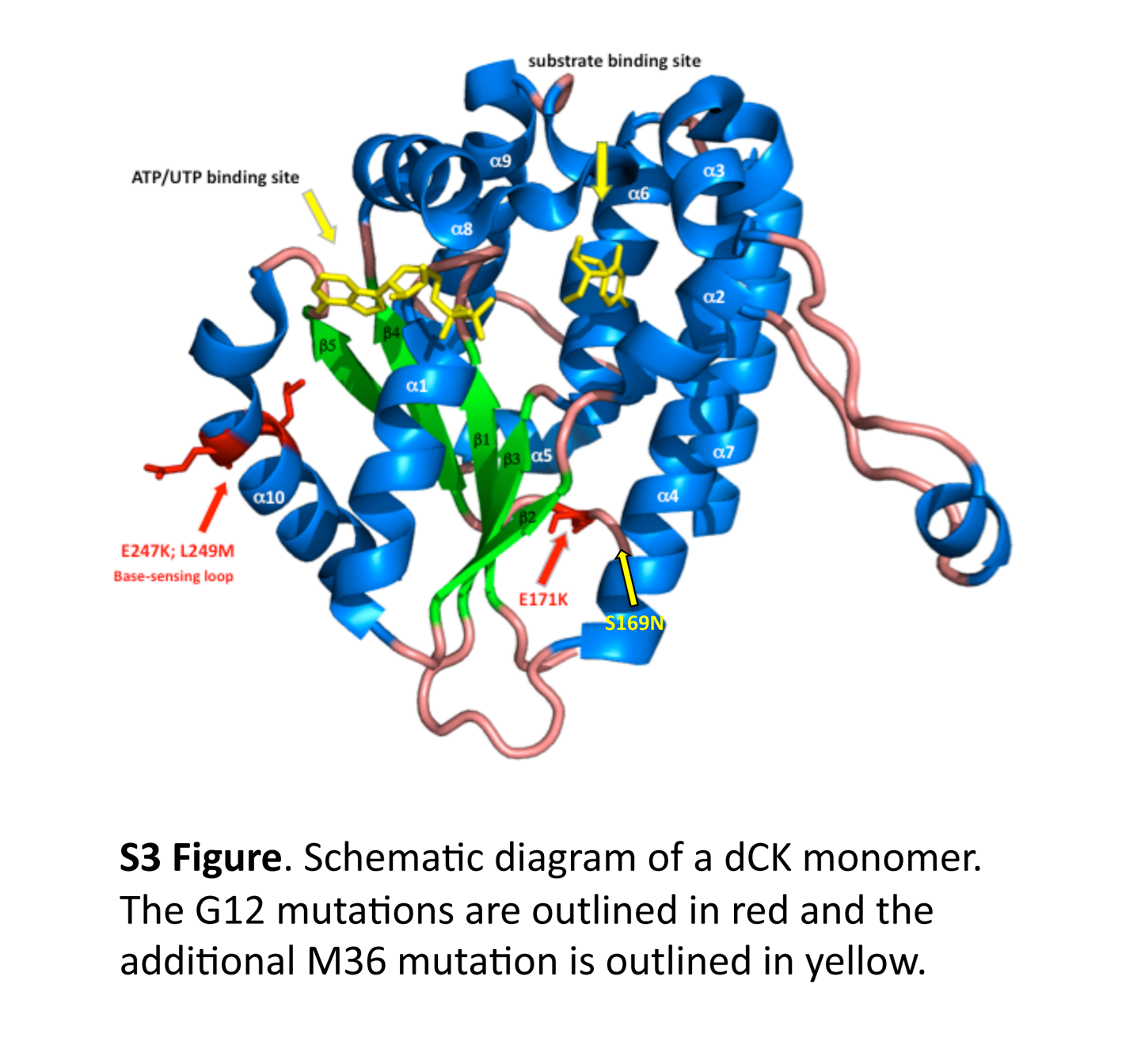

Supplement: S3 Fig — The G12 mutations are outlined in red and the additional M36 mutation is outlined in yellow. (TIF) [file pone.0140741.s003.tif]

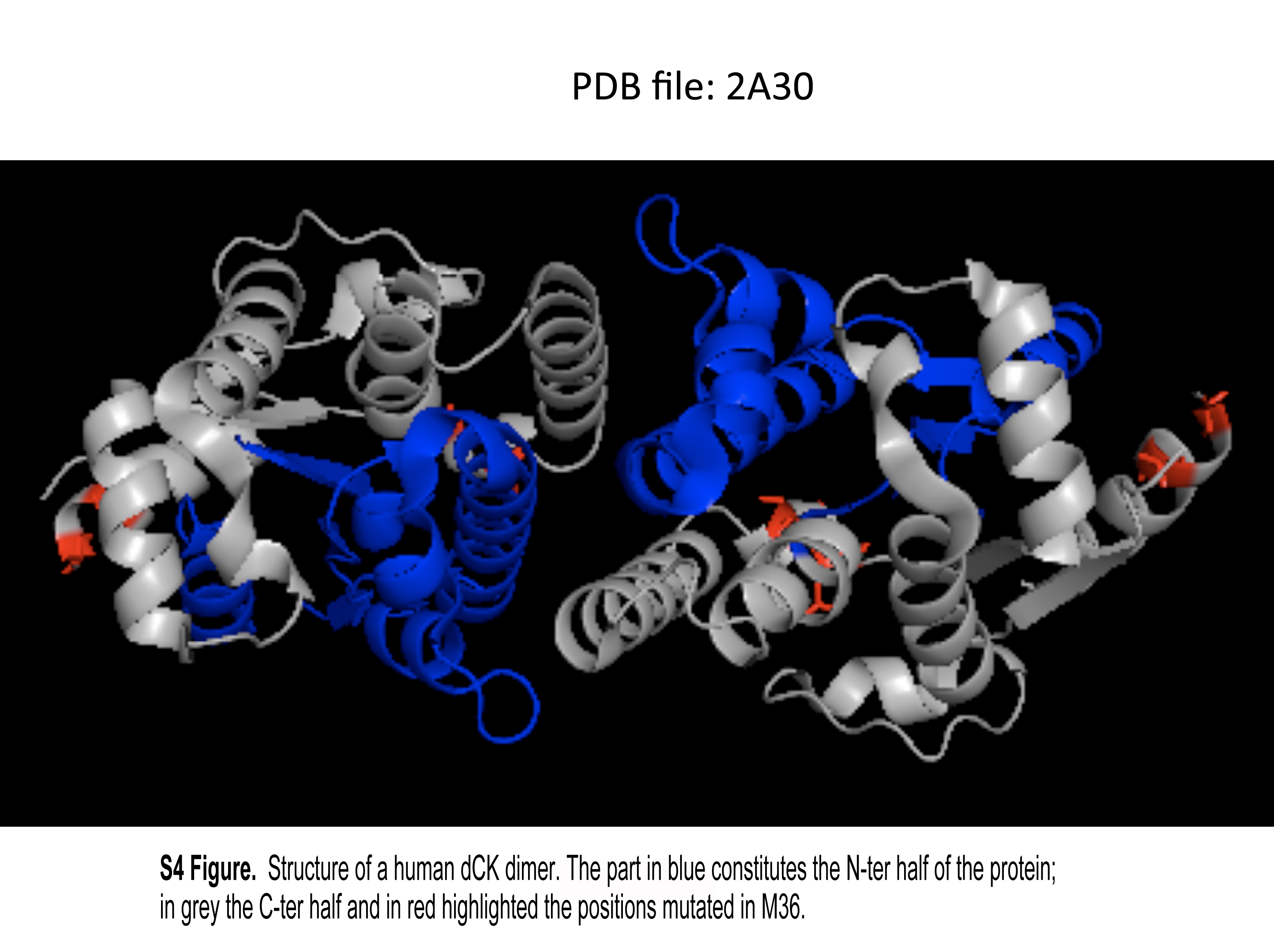

Supplement: S4 Fig — The part in blue constitutes the N-ter half of the protein; in grey the C-ter half and in red highlighted the positions mutated in M36. (TIF) [file pone.0140741.s004.tif]
